# IFN-γ Rα Is a Key Determinant of CD8+ T Cell-Mediated Tumor Elimination or Tumor Escape and Relapse in FVB Mouse

**DOI:** 10.1371/journal.pone.0082544

**Published:** 2013-12-06

**Authors:** Maciej Kmieciak, Kyle K. Payne, Xiang-Yang Wang, Masoud H. Manjili

**Affiliations:** 1 Department of Microbiology & Immunology, Virginia Commonwealth University, Massey Cancer Center, Richmond, Virginia, United States of America; 2 Department of Human & Molecular Genetics, Virginia Commonwealth University, Massey Cancer Center, Richmond, Virginia, United States of America; INRS, Canada

## Abstract

During the past decade, the dual function of the immune system in tumor inhibition and tumor progression has become appreciated. We have previously reported that neu-specific T cells can induce rejection of neu positive mouse mammary carcinoma (MMC) and also facilitate tumor relapse by inducing neu antigen loss and epithelial to mesenchymal transition (EMT). Here, we sought to determine the mechanism by which CD8+ T cells either eliminate the tumor, or maintain tumor cells in a dormant state and eventually facilitate tumor relapse. We show that tumor cells that express high levels of IFN-γ Rα are eliminated by CD8+ T cells. In contrast, tumor cells that express low levels of IFN-γ Rα do not die but remain dormant and quiescent in the presence of IFN-γ producing CD8+ T cells until they hide themselves from the adaptive immune system by losing the tumor antigen, neu. Relapsed tumor cells show CD44+CD24- phenotype with higher rates of tumorigenesis, *in vivo*. Acquisition of CD44+CD24- phenotype in relapsed tumors was not solely due to Darwinian selection. Our data suggest that tumor cells control the outcome of tumor immune surveillance through modulation of the expression of    IFN-γ Rα.

## Introduction

Treatment of primary breast cancer frequently appears to be initially successful; however, the disease may recur either locally or as distant metastases years or even decades later, where the tumor cells have been in a prolonged state of dormancy. In fact, circulating tumor cells that are quiescent have been detected in cancer free patients several years after successful treatment, suggesting that tumor cells are in a state of dynamic dormancy [1,2]. While the basis for tumor dormancy and relapse is poorly understood, there is general agreement that the immune response plays a critical role. 

Tumor immunoediting has a dual function. On the one hand, it inhibits tumor growth and results in the elimination of nascent transformed cells; on the other hand, it edits the tumor resulting in the escape and progression of less immunogenic tumor cells. Bob Schreiber has formulated such opposing effects of the immune system into three phases which include Elimination or tumor rejection, Equilibrium or tumor dormancy, and Escape or cancer formation (3Es) [3]. His group was first to show that adaptive immune response can maintain the tumor in an equilibrium phase or a dormant state, leading to tumor escape [4]. Very recently, it was reported that the innate immune system can also facilitate cancer immunoediting by the production of IFN-γ [5]. These reports suggest a key role for IFN-γ in linking the adaptive and innate immune system during the process of tumor immunoediting.

We have previously shown that neu-specific IFN-γ producing T cells concurrently induced tumor inhibition and tumor escape that led to tumor relapse in FVB mice [6]. Subsequent analysis revealed that such tumor immunoediting was associated with epithelial to mesenchymal transition (EMT) and expression of breast cancer stem cell markers in the relapsed tumor cells [7]. Here, we show that tumor immunoediting is facilitated through IFN-γ/IFN-γ Rα axis such that low, but not high, levels of IFN-γ Rα expression in tumor cells establish tumor equilibrium similar to tumor dormancy, resulting in neu antigen loss and tumor relapse in the presence of IFN-γ producing T cells. 

## Results

### Low expression of IFN-γ Rα in the tumor facilitates tumor escape in the presence of neu-specific CD8+ T cell response

**Figure 1:**
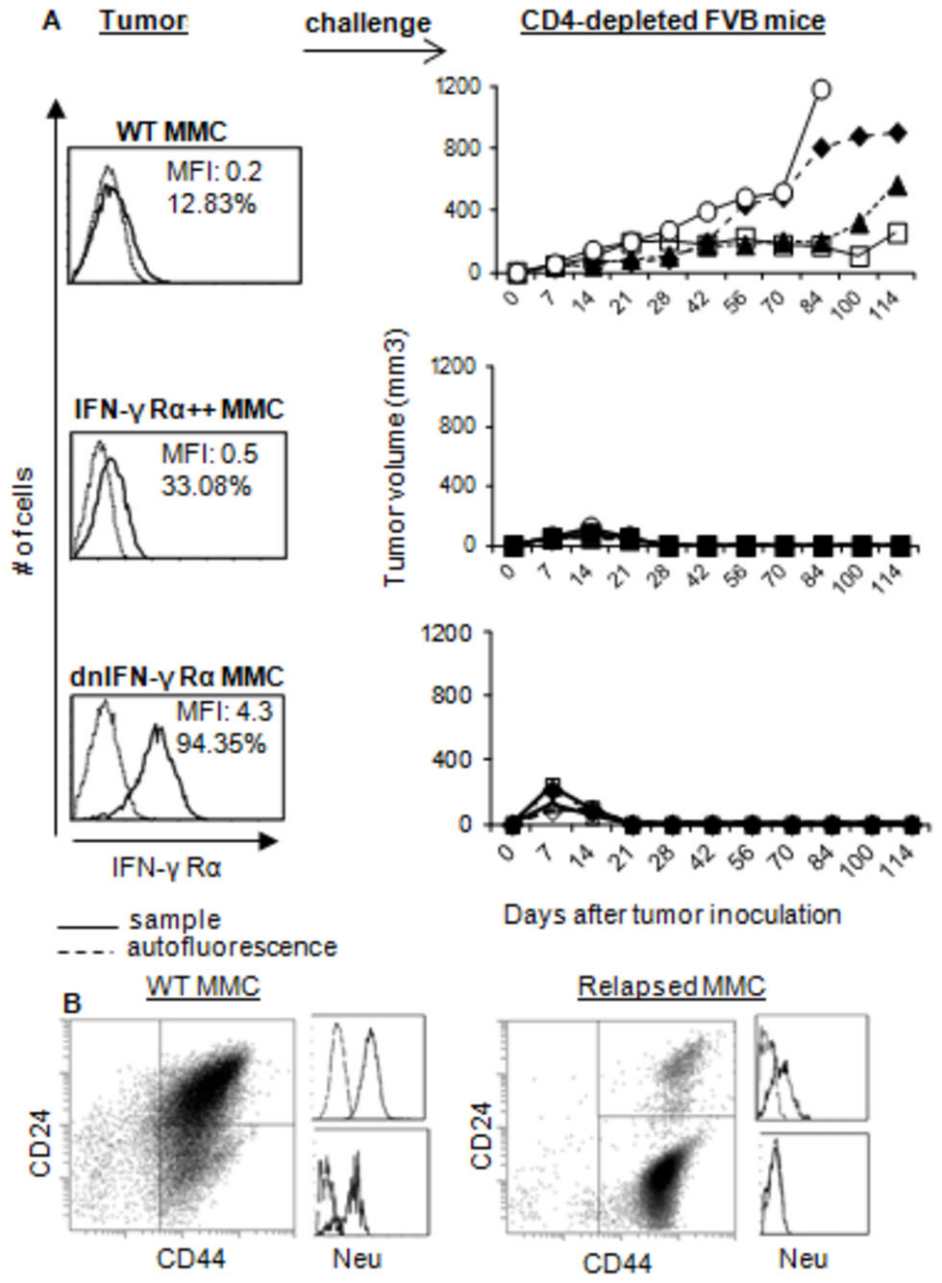
The neu-specific CD8+ T cells facilitate tumor escape and relapse of WT MMC expressing low levels of IFN-γ Rα. WT MMC, IFN-γ Rα++ MMC and dnIFN-γ Rα MMC cells were analyzed for IFN-γ Rα status by flow cytometry and injected (5x10^6^ cells) into CD4-depleted FVB mice (four mice for each group) followed by monitoring tumor growth for 114 days (A). Also, evaluation of CD44+CD24- population and neu expression in WT MMC at the time of tumor challenge (left panel) and after tumor relapse (right panel) by flow cytometry was done (B).

We have previously reported a dual function of neu-specific CD8+ T cell responses in facilitating tumor elimination or tumor escape [6]. Here, we sought to determine whether such opposing effects of CD8+ T cells are mediated through the IFN-γ-IFN-γ Rα axis *in vivo*. To test this, we used FVB mice that naturally mount neu-specific immune responses resulting in complete rejection of neu positive mouse mammary carcinoma (MMC) tumor cells within 3 weeks after challenge [6]. When CD4+ and CD8+ T cells were depleted, FVB mice failed to reject MMC [6]. In order to focus on CD8+ T cells and eliminate the contribution of CD4+ T cells and antibody responses to the tumor rejection, FVB mice were depleted of CD4+ T cells during the course of study. Animals were then inoculated with: i) wild type MMC tumor cell line that normally express low levels of IFN-γ Rα (WT MMC); ii) wild type MMC with forced expression of IFN-γ Rα (IFN-γ Rα++ MMC), or iii) wild type MMC with the expression of dominant negative IFN-γ Rα (dnIFN-γ Rα MMC). As shown in Figure 1A, presence of CD8+ T cells alone failed to reject WT MMC, though it resulted in the cessation of tumor growth for over 2 months; animals eventually succumbed to tumor relapse. All mice (4 mice/group) rejected IFN-γ Rα++ MMC and dnIFN-γ Rα MMC tumor cells. Rejection of dnIFN-γ Rα MMC tumor cells was consistent with our previous observation showing that sorted IFN-γ Rα negative MMC tumor cells were rejected by CD4-depleted FVB mice [6]. We also performed in vivo tumor challenge studies in the presence of GR20 antibody that blocks IFN-γ Rα, showing that WT MMC tumor cells were rejected by CD4-depleted and GR20-treated FVB mice (Figure S1). WT MMC and relapsed MMC tumor cells were then analyzed for the expression of neu and the stem cell markers CD44 and CD24. As shown in Figure 1B, relapsed tumors lost neu expression as well as CD24 expression. During equilibrium phase, tumor cells still had neu expression and stem-like phenotype did not remarkably change compared with WT MMC (data not shown). Both WT MMC and relapsed ANV expressed low levels of IFN-γ Rα (data not shown).

**Figure 2:**
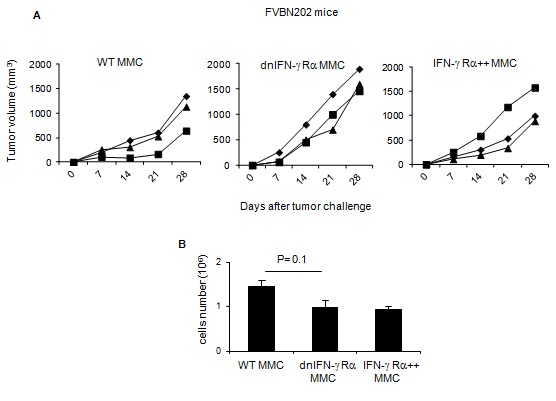
*In* *vivo* and *in* *vitro* proliferation rate of WT MMC, IFN-γ Rα++ MMC and dnIFN-γ Rα MMC cells. 5x10^6^ cells were injected into FVBN202 mice (three repeats) and tumor growth as function of proliferation *in* *vivo* was monitored (A), also tumor cells (0.25x 10^6^/well in triplicates) were cultured for 3.5 days and were counted using trypan blue exclusion (B).

In order to determine if tumor escape or elimination was mediated by neu-specific immune response, the three tumor cell lines were inoculated into FVBN202 mice that tolerate neu positive MMC. All tumor cell lines showed comparable rates of growth *in vivo* within 4 weeks after challenge (p > 0.05), due to the lack of an effective neu-specific T cell response (Figure 2A). All tumor cells also showed comparable rates of proliferation *in vitro*, as determined by trypan blue exclusion (Figure 2B). 

### IFN-γ induces apoptosis and inhibits tumor growth *in vitro*

**Figure 3:**
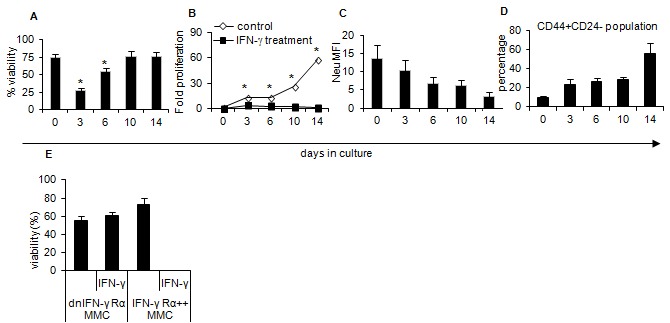
Status of IFN-γ Rα expression in the tumor cells determines the type of response to IFN-γ. WT MMC tumor cells were cultured with IFN-γ (50 ng/ml/10^6^ cells) for two weeks and were analyzed at different time points for viability (Annexin V-/PI-) by flow cytometry (A), cell proliferation by trypan blue exclusion (B), neu expression (C) and evaluation of CD44+CD24- population (D) by flow cytometry. E) dnIFN-γ Rα MMC or IFN-γ Rα++ MMC were also culture in the absence or presence of IFN-γ (50 ng/ml/10^6^ cells) for 3 days and percent viable cells were counted by trypan blue exclusion. Data represent 2-3 independent experiments.

To determine whether IFN-γ may be responsible for tumor escape and relapse of neu antigen negative variant (ANV) from primary MMC tumors, WT MMC tumor cells, dnIFN-γ Rα MMC or IFN-γ Rα++ MMC were cultured with IFN-γ (50 ng/10^6^ cells/ml). IFN-γ was added with fresh medium once every 3 days and proliferation of adherent cells, i.e. viable cells was determined while floater cells were excluded. As shown in Figure 3A, IFN-γ induced apoptosis in the majority of WT MMC cells within the first 6 days of culture (p < 0.02), thereafter continuous supply of IFN-γ into the culture failed to induce apoptosis such that all tumor cells remained viable. Compared with control MMC tumor cells, IFN-γ treatment resulted in significant inhibition, but not a complete cessation, of tumor cell proliferation on days 3 (p=0.011), 6 (p=0.016), 10 (p=0.015) (Figure 3B). Flow cytometry analysis showed downregulation of the neu expression from an average MFI of 13.6 to 3.25 on tumor cells within the first 14 days of culture with IFN-γ (Figure 3C). We have already shown that such downregulation of the neu expression was associated with methylation of the neu promoter [6]. IFN-γ also induced downregulation of CD24 expression such that CD44+CD24- stem-like MMC tumor cells were increased from an average 10% to 55% (Figure 3D). The removal of IFN-γ from the culture restored proliferation of tumor cells as well as the expression of neu and CD24 within 2 weeks (data not shown). As expected, the presence of IFN-γ did not induce apoptosis in dnIFN-γ Rα MMC, though all IFN-γ Rα++ MMC cells died in the presence of IFN-γ (Figure 3E). 

### IFN-γ inhibits proliferation of CD44+CD24+ and CD44+CD24- tumor cells

**Figure 4:**
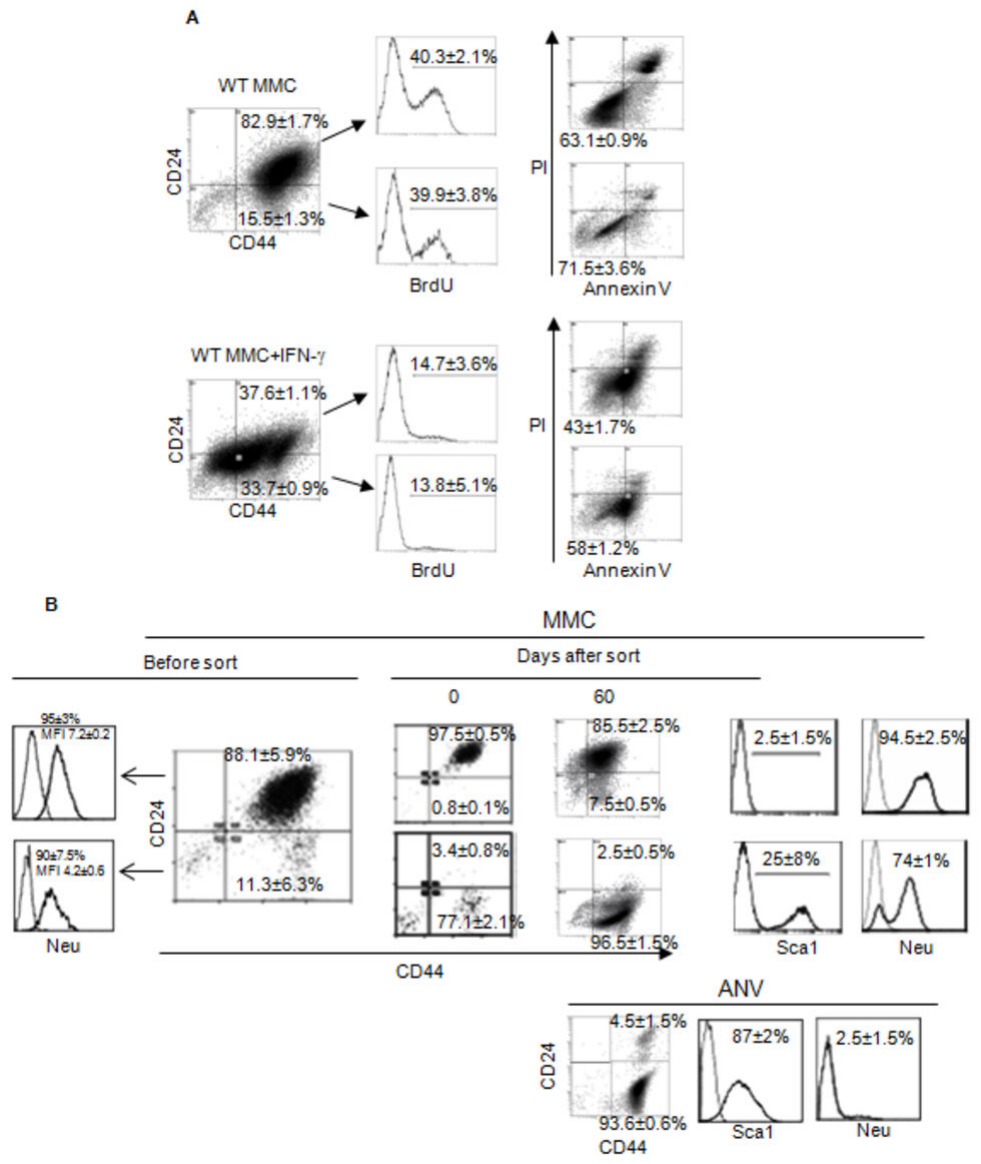
The CD44+CD24- stem-like population and CD44+CD24+ population of WT MMC respond similarly to IFN-γ. WT MMC tumor cells were cultured with or without IFN-γ (50 ng/ml/10^6^ cells) for 3 days and CD44+CD24- as CD44+CD24- populations were analyzed for viability (Annexin V-/PI-) and proliferation (BrdU) by flow cytometry (A), these two population where sorted from WT MMC cells using a BD FACSAria III cell sorter and cultured for 60 days, after that analysis of CD24, CD44, Sca1 and neu was done (B) by flow cytometry. Also analysis of relapsed ANV tumor is shown. Data represent two independent experiments.

In order to determine if IFN-γ induces apoptosis in CD44+CD24+ cells and select for CD44+CD24- cells within WT MMC, we determined proliferation as well as apoptosis in each subpopulation within WT MMC using multi-color flow cytometry analysis. As shown in Figure 4A, IFN-γ treatment exhibited comparable effects on the inhibition of cell proliferation. The BrdU staining showed a 3-fold inhibition of cell proliferation in CD24+ and CD24- cells after IFN-γ treatment. Also, Annexin V/PI staining showed comparable decreases in the viability of CD24+ and CD24- cells after a 3-day culture with IFN-γ (Figure 4A, 63% to 43% vs. 72% to 58%). Together, these data suggest that the effect of IFN-γ on WT MMC is not simply Darwinian selection of the CD44+CD24- subpopulation. To further explore this, WT MMC were sorted into CD44+CD24+ and CD44+CD24- subpopulations in order to determine whether CD44+CD24- subpopulation within MMC could expand into a neu negative phenotype similar to ANV. As shown in Figure 4B, both subpopulations expressed neu protein before sorting. Sorted cells were then cultured *in vitro* in the absence of IFN-γ for 2 months. Unlike ANV, CD44+CD24- MMC cells retained the expression of neu throughout the *in vitro* culture; they also retained CD44+CD24- phenotype with the expression of the stem cell marker Sca1. Sorted CD44+CD24+ cells established a cellular phenotype similar to WT MMC with 8% CD44+CD24- cells.

### MMC tumor cells contain CD44+CD24- stem-like cells

**Figure 5:**
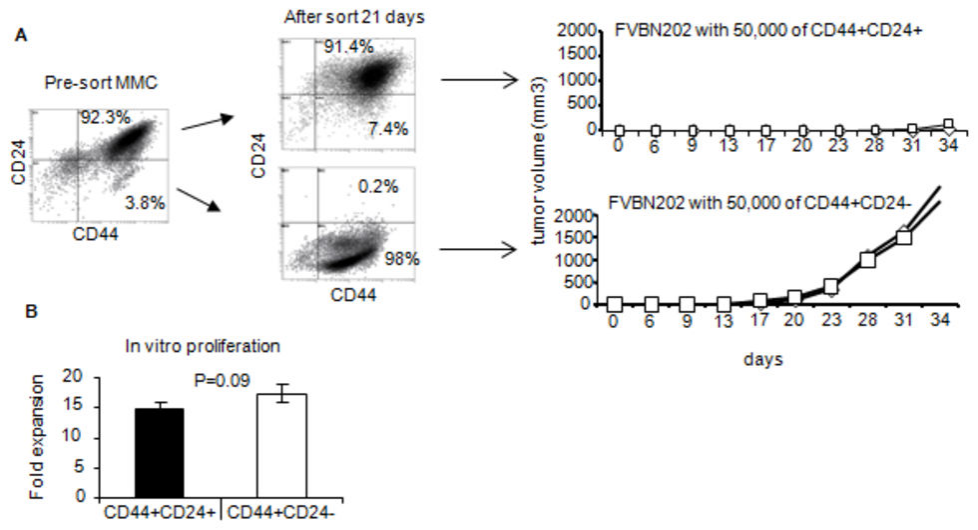
CD44+CD24- stem-like tumor cells show greater tumorigenicity compared with CD44+CD24+ population of WT MMC. WT MMC tumor cells were sorted into CD44+CD24- and CD44+CD24- populations and on day twenty one, 50,000 of the sorted cells were injected into mice (n=2) and tumor growth was observed. Sorted tumor cells were also cultured for 10 days (in triplicates) and counted using trypan blue exclusion. Fold of expansions are shown (B).

Since CD44+CD24- breast cancer cells have been suggested to be cancer stem-like cells which also express the stem cell marker Sca1, we sought to determine the stemness capacity of the sorted cells. FVBN202 transgenic mice were inoculated with a low dose of sorted CD44+CD24+ or CD44+CD24- MMC (50,000 cells/mouse). As shown in Figure 5A, sorted CD44+CD24+ cells failed to establish large tumors within 3-4 weeks after challenge, whereas animals succumbed to the tumor within 4 weeks after challenge with sorted CD44+CD24- cells. No appreciable differences were observed in the proliferation of sorted CD44+CD24+ and CD44+CD24- MMC *in vitro* (Figure 5B). We also inoculated FVBN202 mice with a low dose of relapsed ANV on the right side and with WT MMC on the left side showing that ANV tumor cells were more tumorigenic than WT MMC tumor cells (Figure S2). 

## Discussion

We have previously reported that neu tumor antigen loss could occur in the presence of robust neu-specific immune responses in FVB mice leading to tumor relapse of the neu antigen negative variant, ANV [6]. We have also shown that CD8+ T cells were involved in the epithelial to mesenchymal transition (EMT) associated with neu antigen loss and tumor relapse [7]. Here, we determined that neu-specific CD8+ T cells induce tumor relapse through the IFN-γ-IFN-γ Rα axis. The level of IFN-γ Rα expression on tumor cells was found to be a key predictor of responsiveness of the tumor to CD8+ T cells. High levels of IFN-γ Rα expression resulted in T cell-mediated tumor rejection and relapse-free survival whereas low levels of IFN-γ Rα expression facilitated CD8+ T cell-induced tumor inhibition and retention of tumor equilibrium, leading to tumor relapse. Rejection of dnIFN-γ Rα MMC by CD4-depleted FVB mice was consistent with our previous observation showing that sorted IFN-γ Rα negative MMC tumor cells were rejected by CD4-depleted FVB mice [6]. This rejection could be due to IFN-γ-independent mechanisms such as perforin/granzyme, which is more active in the absence of IFN-γ signaling. We observed that IFN-γ can induce expression of serine protease inhibitor 6 (SPI6) in WT MMC whereas dnIFN-γ Rα MMC did not express SPI6, thus remaining susceptible to granzyme B-mediated apoptosis (unpublished data). SPI6 has been shown to block granzyme-induced apoptosis [8,9], thereby inhibiting IFN-γ-independent pathway of tumor rejection in tumor cells that express low levels of IFN-γ Rα. Relapsed ANV tumor cells showed characteristics of stem-like cells which included CD44+CD24- phenotype, Sca1 expression, and high rates of tumorigenicity *in vivo*. Finally, phenotype analysis by cell sorting suggested that neu antigen loss and tumor relapse were not merely due to Darwinian selection. These findings are consistent with our previous reports on T cell-mediated induction of EMT and IFN-γ-induced methylation of the neu promoter [6,10].

Quiescent breast cancer cells have been detected in the circulation of patients up to 22 years after diagnosis and successful treatment [1]. These observations suggest that tumor cells can remain in the state of dormancy, raising concerns not only for breast cancer survivors but also for cancer free individuals who may carry quiescent malignant cells without clinical symptoms of cancer. Therefore, understanding the mechanisms of tumor dormancy and recurrence in immunocompetent individuals may lead to a breakthrough in the treatment and elimination of breast cancer. Here, we showed that an effective CD8+ T cell response can facilitate tumor equilibrium which may mimic tumor dormancy in tumor clones that expressed low levels of IFN-γ Rα. Our finding is consistent with previous reports in the murine B cell lymphoma model, demonstrating that IFN-γ producing CD8+ T cells, but not helper T cells, were required for the establishment and maintenance of tumor dormancy in the immunized mice [11]. Using a mouse model of primary chemical carcinogenesis, Koebel et al. [4,12] also demonstrated that IFN-γ producing T cells facilitate tumor dormancy by inhibiting proliferation of tumor cells *in vivo*. They also showed that tumor cells in dormant state or equilibrium are unedited but become edited when they spontaneously escape immune control and grow into clinically apparent tumors. IFN-γ was found to play a key role in tumor escape by downregulating the expression of NKG2D ligand on tumor cells [13,14], selecting for tumor clones with reduced immunogenicity [15], and downregulating the tumor antigen [6,16-19]. Very recently, DuPage et al. [17] adapted a genetically engineered mouse model of sarcoma in order to investigate the process of cancer immunoediting. They found that primary sarcomas were immunoedited to become less immunogenic and escape immune surveillance by losing tumor antigen expression or presentation on MHC class I [17]. In patients with ductal carcinoma in situ (DCIS), HER-2/neu-targeted vaccination induced HER-2/neu-specific IFN-γ producing T cell responses and resulted in the inhibition of DCIS as well as the induction of HER-2/neu antigen loss [18,19]. In fact, IFN-γ producing T cells are involved in tumor immune surveillance to inhibit primary tumors as well as in tumor immunoediting to shape tumor dormancy, leading to tumor escape by losing the target antigen in immunologically intact individuals. We have previously reported variations in tumor IFN-γ Rα expression in human breast tumor specimens [20], suggesting a potential variation in the tumor responsiveness to the immune system. It has been reported that pro-inflammatory cytokines can modulate the expression of IFN-γ Rα [21], again suggesting a link between inflammation and tumor immune editing.

Dormant tumor cells are linked to the acquisition of cancer stem-like characteristics. It has been well established that breast cancer stem cells are characterized by CD44+CD24- phenotype, expression of Sca1 with high rates of tumorigenicity, *in vivo* [22-26]. Our data suggest that relapsed tumor cells, ANV, show characteristics of breast cancer stem-like cells. This is consistent with a recent report showing that the CD44+CD24- phenotype contributes to breast cancer relapse [23]. There was no correlation between stem-like cells and levels of IFN-γ Rα expression, because ANV cells showed low levels of IFN-γ Rα expression. Also, in WT MMC cells with heterogeneity in the expression of IFN-γ Rα ranging from negative to low expression, levels of IFN-γ Rα expression did not correlate with stem-like cells (data not shown). However, ANV cells were not able to generate CD44+CD24+ primary MMC tumor cells, *in vitro*. In addition, phenotype analysis of CD44+CD24+ and CD44+CD24- populations from within primary MMC tumor cells suggest that relapsed ANV tumor cells did not originate from the CD44+CD24- phenotype as a result of immune selection; unlike ANV cells, the CD44+CD24- phenotype of MMC retained neu expression during *in vitro* culture. These findings are consistent with our previous observation that neu antigen loss was due to epigenetic modification resulting in the hypermethylation of the promoter region of the *neu* gene [6]. Retention of CD44+CD24- phenotype during *in vitro* and *in vivo* passages suggest that CD44+CD24-Sca1+ cells may be a highly tumorigeneic phenotype within the primary tumor cells which have characteristics similar to stem-like cells, but they may not be tumor initiating cells.

Here, we showed that tumor cells, but not the immune response, can determine the outcome of anti-tumor adaptive immune responses through modulation of the expression of IFN-γ Rα. In a recent review article, we discussed both preclinical and clinical evidence that highlight the opposing function of IFN-γ producing T cells in tumor inhibition and tumor escape or relapse [27]. Our data also suggest that CD8+ T cells can facilitate tumor relapse through IFN-γ Rα which result in the acquisition of stem-like phenotype. The genetic complexity and heterogeneity of cancer is becoming increasingly appreciated through genomic and histological analyses. In fact, heterogeneity of breast cancer cells in the levels of IFN-γ Rα expression suggests selective induction of tumor dormancy only in the clones that express low levels of IFN-γ Rα. Therefore, it would be critical to target these clones in order to overcome tumor relapse.

## Methods

### Mice

Wild-type FVB (Jackson Laboratories) or FVBN202 transgenic (Charles River Laboratories) female mice (6–12 weeks of age) were housed under aseptic conditions and used throughout these studies. The studies have been reviewed and approved by the Institutional Animal Care and Use Committee (IACUC) at Virginia Commonwealth University.

### Depletion of lymphocytes

Depletion of CD4 was performed by i.p. injections of GK1.5, as previously described by our group [7]. Briefly, animals were injected with the GK1.5 (200–250 µg/mouse) on days -2 and -1 prior to tumor challenge followed by the injections once every 5 days until the completion of the trial. Depletion of the lymphocyte subsets was assessed on the day of tumor challenge and bi-weekly thereafter by FACS analysis of peripheral blood. Blockade of IFN-γ Rα was performed by the i.p. injection of GR20 antibody (200 µg/200 µl/mouse) prior to tumor challenge and once every 3 days after tumor challenge.

### Tumor cell lines

The neu overexpressing mouse mammary carcinoma (WT MMC) cell line was established from the spontaneous mammary tumors harvested from FVBN202 mice, as previously described by our group [6]. The neu antigen-negative variant (ANV) tumor cell line was derived from a relapsed MMC tumor under the neu-specific immune pressure, as previously described by our group [6]. The dnIFN-γ Rα and IFN-γ Rα vectors were gifts from Dr. William Lee of the U Penn. We established IFN-γ Rα++ MMC cell line by stable transfection of pcDNA3 vector (Invitrogen) containing mouse IFN-γ Rα ORF construct [7]. For this, cDNA of IFN-γ Rα was amplified using mouse mammary tumor cDNA library as a template in PCR reaction with proofreading polymerase (Accuzyme, BioLine). PCR conditions were as follows: 94°C 3 min, 94°C 30 s, 48°C 30 s, 68°C 2 min (10 cycles); 94°C 30 s, 60°C 30 s, 68°C 2 min (20 cycles) followed by 10 min extension at 68°C. Primers used in this reaction: sense: 5’-TTTATGGTACCATGGGCCCGCAGGCGGCA-3’ and antisense: 5’-TTAGATATCTTAGGACAGCTCCTGGGCCTC-3’ containing restriction sites for KpnI and EcoRV, respectively (underlined). Amplified cDNA fragment and pcDNA3+ vector (Invitrogen) were then digested with KpnI and EcoRV endonucleases (NEB BioLabs) for preparing compatible ends for ligation reaction. After ligation, constructs with insert were isolated and sequenced to confirm intact expression of the gene. Transfection of MMC cells with construct was done using Lipofectamine 2000 Reagent (Invitrogen) according to manufacture protocol. Tumor cells were maintained in RPMI 1640 supplemented with 10% FBS. We used G418 antibiotic at a 200 µg/ml (Gibco) for the selection.

### In vivo tumor challenge

Female mice were inoculated s.c. with the tumor cell lines as previously described by our group [7]. Animals were inspected twice every week for the development of tumors. Tumor masses were measured using calipers along the two perpendicular diameters. Tumor volume was calculated by: V (volume) = L (length) x W (width)^2^ / 2. Mice were killed before a tumor mass exceeded 2,000 mm^3^.

### IFN-γ treatment

Adherent tumor cells were incubated with 50ng/ml of IFN-γ (Biolegend) for 3 days. After a 3-day culture, cells were washed and added with fresh medium containing IFN-γ every 5-7 days.

### Flow cytometry

A three color staining flow cytometry analysis of the mammary tumor cells (10^6^ cells/tube) was carried out as previously described by our group [7]. Fc blocking was performed using anti-CD16/CD32 Ab (Biolegend). For Annexin V staining, cells were stained for respective surface markers, washed with cell staining buffer followed by washing with 1x Annexin V buffer (BD Pharmingen). The Annexin V staining protocol was then performed. We used following antibodies: mouse anti-neu (Ab-4) (Calbiochem, San Diego, CA), isotype control Ig, PE or FITC conjugated anti-mouse Ig (Biolegend), FITC-conjugated Annexin V and propidium iodide (PI) (BD Pharmingen, San Diego, CA), PE/Cy5-CD24, FITC-CD44, PE-CD44, PE-Sca-1, PE-IFN-γ Rα and isotype Igs (Biolegend). All Abs were used at the manufacture’s recommended concentrations. Multicolor data acquisition was performed on a BD FACSCanto II and analyzed using BD FACSDiva software. Cell cycle staining was done with standard protocol for staining EtOH fixed cells with PI.

### BrdU staining

FITC BrdU Flow Kit (BD Pharmingen) was used in proliferation assays as described previously [21]. 

### Cell sorting

To sort distinct cellular populations of WT MMC, 10^7^ cells were added to sample tubes in which FcRs were blocked and surface markers were stained with PE-conjugated CD44 and PE/Cy5-conjugated CD24 Abs. Cells were then washed with sterile PBS supplemented with 2% FBS. The CD44+CD24+ and CD44+CD24- populations were sorted into 100% FBS using a BD FACSAria III cell sorter. 

### Statistical analyses

Graphical data are presented as means with standard errors. Statistical comparisons between groups were made using Student’s *t* test with P< 0^.^05 being statistically significant. 

## Supporting Information

Figure S1 **Blockade of IFN-γ Rα in vivo result in CD8+ T cell-mediated rejection of WT MMC.** CD4-depleted FVB mice (n=2) were injected i.p. with GR20 antibody and then inoculated with WT MMC tumor cells (3x10^6^ cell/mouse). Animals received GR20 antibody once every three days and tumor growth was determined.
(PDF)

Figure S2 **ANV tumor cells are more tumorigenic than WT MMC tumor cells.** FVBN202 mice (n=4) were inoculated with a low dose (60,000/mouse) ANV tumor cells on the right side and WT MMC tumor cells on the left side. Tumor growth was monitored.
(PDF)
