# Supplementary figures and images for: IFN-γ Rα Is a Key Determinant of CD8+ T Cell-Mediated Tumor Elimination or Tumor Escape and Relapse in FVB Mouse
Source: PLoS One. 2013 Dec 6;8(12):e82544. doi: 10.1371/journal.pone.0082544 (PMC3855782; doi:10.1371/journal.pone.0082544)

**Figure S1**

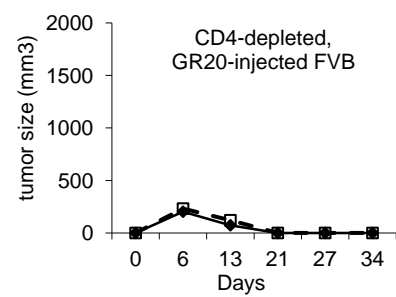

Supplement: Figure S1 — Blockade of IFN-γ Rα in vivo result in CD8+ T cell-mediated rejection of WT MMC. CD4-depleted FVB mice (n=2) were injected i.p. with GR20 antibody and then inoculated with WT MMC tumor cells (3x10^6^ cell/mouse). Animals received GR20 antibody once every three days and tumor growth was determined. (PDF) [file pone.0082544.s001.pdf]

Figure S5

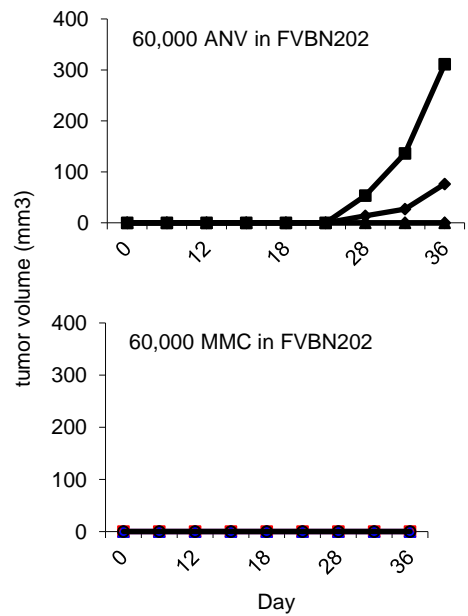

Supplement: Figure S2 — ANV tumor cells are more tumorigenic than WT MMC tumor cells. FVBN202 mice (n=4) were inoculated with a low dose (60,000/mouse) ANV tumor cells on the right side and WT MMC tumor cells on the left side. Tumor growth was monitored. (PDF) [file pone.0082544.s002.pdf]
